# Meditation as a Therapy or a Threat: A Case Series

**DOI:** 10.7759/cureus.75201

**Published:** 2024-12-06

**Authors:** Amrendra K Singh, Umesh Pathak, Partik Kaur, Rashmi Dhakad

**Affiliations:** 1 Department of Psychiatry, Atal Bihari Vajpayee Government Medical College, Rewa, IND; 2 Department of Psychiatry, Birsa Munda Government Medical College, Shahdol, Shahdol, IND; 3 Department of Psychiatry, MGM Medical College, Indore, Indore, IND; 4 Department of Psychiatry, Gandhi Medical College, Bhopal, Bhopal, IND

**Keywords:** acute psychosis, mania, meditation, mindfulness, pyschosis

## Abstract

Meditation is an approach to self-regulate the emotions. Meditation has a beneficial effect on both physical and mental health. Various forms of meditation practices prevail in many religions and cultures for human well-being. Although meditation has so many positive effects, very few people experience negative too. Meditation-induced psychosis has been reported in the past. Here, we present a case series of seven cases who developed psychiatric illness after meditation.

## Introduction

Meditation has been widely practiced for many years as a way to promote a healthy state and for religious purposes. In recent years, meditation has been used as a general term to refer to various practices aimed at self-regulation of thoughts and emotions [1]. Its effects on the mind and body are now being studied and used in medicine. Meditation predominantly has beneficial effects on the body and mind. In past years, meditation has been used as a common term to refer to various types of practices for the regulation of thoughts by self [2]. Based on the assumption that different neurophysiological states accompany different states of consciousness, the neuroscientific approach focuses on changing emotions, cognition, and self-awareness. Meditation-induced neurophysiological changes occur in the body and mind [3].

Mindfulness meditation has been successfully integrated with psychotherapy, such as dialectical behavior therapy [4]. It is also regarded as beneficial in various psychiatric illnesses like anxiety disorders, mood disorders, sleep disorders, attention-deficit/hyperactivity disorder, substance use disorders, and suicidality. However, excessive meditation might result in psychiatric manifestations, although the data available is very low. Retreat courses or long durations of intense meditation for many times coupled with practices of prolonged fasting, sleep dysregulation, sensory deprivation, A-sociality, and intense efforts to get inner consciousness can contribute to inducing psychosis after meditation [5-8].

There is ample literature on the benefits of meditation, including mindfulness-based practices. However, evidence is limited on the potential harms. Only recently have academic institutions and doctors who specialize in the study of meditation-based therapy started to recognize that some people may have negative or harmful consequences after meditating [9]. However, it is equally important to study the risks associated with meditative practices, like the adverse effects of pharmacotherapy. The knowledge of the possible harm due to meditation could help in improving its effectiveness. With this aim, we are reporting cases where the symptoms were mostly preceded by some sort of meditation practice.

## Materials and methods

This is a case series formulated after observation of patients with psychiatric manifestations who have a prior history of intense meditation just before the onset of symptoms. We have taken seven cases from our OPD with a history of intense meditation before the symptoms and formulated a case series showing the impact of intense meditation on their mental health. Informed consent was taken from the patients. The report has been prepared according to the case report guideline (CARE checklist; see Appendix) [10].

Case presentation

Case 1

A 26-year-old Hindu female student, presented in the OPD with symptoms of increased physical activity, overfamiliarity, increased talk, and overgrooming for 15 days. The mental status examination (MSE) shows elevated mood and increased self-esteem. These symptoms started after attending a center for meditation for four months, where they guided her about the world and shared their views. Initially, she used to talk excessively about their views of the world and practice meditation two times a day. With time, she started doing meditation at any time in the day, for hours, and spent more time at the center. Initially, she became irritable with friends and family. She tried to make new friends, started talking with strangers, and frequently went to parties. She feels that she is special and can do anything through the vibes generated from meditation. These symptoms gradually developed over a month. It was an unguided and intense session of mediation she used to do randomly at any place. There was no fasting although sleep deprivation was present. She had no history of substance abuse and no contributory family history and was premorbidly well-adjusted. She had no history of any medical illness, trauma, or past psychiatric history. Psychiatric examination showed increased self-esteem, euphoric mood, and ideas of grandiosity. Physical examinations and laboratory investigations showed no abnormalities. Neuropsychological testing disclosed no cognitive deficits. The patient was diagnosed with mania and started on lithium 750 mg in divided doses, and the patient had also stopped meditation and visits to the center. After stopping meditation and with lithium 750 mg, she improved in seven days and continued the same treatment for six months.

Case 2

A 30-year-old Hindu female postgraduate bank employee joined a meditation and spirituality center. After a few months, she left her job and home and shifted permanently to that meditation center. Slowly, over two years, her behavior started changing. She had decreased communication. She was suspicious that the people in her center were planning a conspiracy against her. She started hearing her thoughts and the voices of people around her. She started feeling like she was going to marry a guy in the center. Due to all these symptoms, the higher authority at the meditation center called her parents. Her parents brought her back home and consulted in the psychiatry department OPD. In the OPD, she presented with complaints of hearing voices, suspiciousness, and decreased sleep. She was premorbidly well-adjusted. She had no history of any drug abuse, medical illness, trauma, or past psychiatric history, and her family history was nil contributory. On MSE, her psychomotor activity was reduced, with blunt affect and restricted mobility, and she had delusions of persecution and second-person and third auditory hallucinations. Her insight was grade 2. She was diagnosed with schizophrenia and started on olanzapine 10 mg, which is hiked up to 20 mg in seven days. Her symptoms showed improvement on 20 mg in 15-20 days, and she continued on the same treatment and was discharged. She was continued on 20 mg of olanzapine for six months, and then the dose was reduced to 15 mg and continued.

Case 3

A 27-year-old unmarried male medical science student was preparing for post-graduation and belongs to the upper-middle-class socioeconomic status. The patient went to the ENT OPD with a complaint of heaviness in the forehead and nose with difficulty in breathing in the morning. After a careful examination, the doctor prescribed him some medication for nasal congestion and told him not to worry. However, despite many visits to the ENT OPD and all investigations, his symptoms were not improved. His friend advised him to do meditation, and he started the same. Initially, he used to meditate for 10 minutes, gradually meditating twice a day, each session lasting one hour. He also reduced his diet. In the beginning, he felt some congestion and heaviness, and he took treatment but was not relieved. He stopped medication prescribed by the ENT department and gradually intensified meditation, and the symptoms of congestion reduced. After a duration of six months, he suddenly started believing that someone was controlling his breathing, became suspicious about friends, became irritable, and decreased social interaction. His sleep was decreased. After 15 days, he was brought up in the OPD by his roommate and diagnosed with acute and transient psychotic disorder (ATPD). He was premorbidly well-adjusted, doing regular physical workouts. He had no history of any substance abuse, medical illness, trauma, or past psychiatric history, and his family history was not contributory. The patient was prescribed a tablet of olanzapine 10 mg HS and lorazepam 2 mg HS for 10 days and called for follow-up. His sleep was normalized, and improvement in other symptoms was also seen. Lorazepam was tapered and stopped. He was given olanzapine 10 mg for 30 days and called for a follow-up. In the next follow-up, the symptoms were resolved, and olanzapine was tapered and stopped in the next two years.

Case 4

A 43-year-old Hindu male, graduate and shopkeeper by occupation, is married and lives with his family. He had a history of one episode of mania three years ago and was stable on treatment. Her wife brought him into OPD with symptoms of increased talk, increased physical activity, decreased sleep, and overfamiliar behavior. Before the onset of symptoms, the patient joined a residential meditation program. It was a 10-day residential course in which the person was involved in meditation for 11 hours per day. After coming from that program, his family noticed some behavior changes. He started talking excessively. His physical activities increased, and he did not go to sleep at night. He started talking to unfamiliar people in parks and public places. On MSE, he has raised psychomotor activity, overfamiliarity, elevated mood, and pressured speech. However, he did not stop treatment and continued it during the program. He has no history of substance intake, and his urine drug test is also negative. He was admitted for further management. His sodium valproate was increased to 1500 mg in divided doses with olanzapine 10 mg with injection of haloperidol 10 mg and promethazine 50 mg. After five days, injectables were removed, and olanzapine was hiked to 15 mg and sodium valproate 1750 mg in divided doses. The patient improved in 10 days and was discharged on oral medication. 

Case 5

A 38-year-old Hindu widowed female, a graduate social worker, was brought to the emergency department with complaints of sudden fainting and falling on the ground for one hour. The patient’s relative gave a history of social withdrawal, poor interaction with people, self-muttering, disturbed sleep, and poor oral intake for the past five days. As per history, she had lost her husband three years ago in an accident, and she had no children, but overall, her premorbid personality was well-adjusted. She recently joined the ashram one month ago, and the symptoms emerged after the practice of deep and intense meditation for the past seven days at the ashram. She started remaining detached from people. Her interaction with people decreased. Her food intake and sleep gradually decreased, and she started getting more and more involved in deep meditation. She stopped taking food for the past two days, stating that she does not require food or water. On a detailed MSE, her affect was shallow, and nihilistic delusions were found. She reported that her body was non-existent, her organs were not functioning, and she was already dead. She refused to eat because of the feeling of being dead. She also reported that she could have conversations with spirits. There was no history of medical, surgical, or psychiatric illness in the past. There was also no history of any substance abuse or stressful events and no family history. On examination, there was no significant abnormality except for low blood pressure. Her neurological examination was normal. Her initial routine blood investigations, urine toxicology, ECG, cardiac 2D echo, CT brain, and EEG did not show any significant abnormalities except a low glucose level (blood sugar 60 mg/dl). She was diagnosed with an acute and transient psychotic disorder and was kept in the medical ward. She recovered with oral olanzapine (5 mg/d) and hiked up to 10 mg; within five to seven days, she showed improvement. Olanzapine was stopped after 15 days because of increased RBS and shifted to risperidone 3 mg, and she was advised not to do intense meditation without proper instruction. She never relapsed in subsequent follow-ups of one year, even after the cessation of psychotropics.

Case 6

A 21-year-old Hindu male came to psychiatry with episodes of “not feeling himself," “as if outside his body,” and “feeling of being detached from the body" for the past month. These experiences were accompanied by feelings of an unreal or automated self and surroundings. He felt that his body seemed lifeless and detached and that their surroundings seemed to lack color and life and appeared artificial or as a stage on which people were acting contrived roles. There was no history of substance use or psychiatric illness in the past or in the family. The medical and surgical history was insignificant for symptoms. There were no stressors or conflicts. The patient had a well-adjusted and stable personality. The only change in his life was that he had started meditation from random YouTube videos on weekends. He experienced these episodes after every meditation practice on the weekends. He was worried about the recurrence of such episodes, which were causing significant impairment in his functioning. On investigations, no medical or organic etiology was identified. The physical examination was "unremarkable," including a normal neurological function. The patient was given a provisional diagnosis of depersonalization-derealization syndrome. He was instructed to stop unguided meditation, and fluoxetine (20 mg/d) with clonazepam (0.25 mg/d) twice daily dose was given for 10 days. The episodes stopped and did not occur on next week. He was followed up on a monthly basis, and three months later, clonazepam was stopped after tapering. He had no further episodes, even after stopping the treatment.

Case 7

A 44-year-old male, high school pass and shopkeeper, was brought to the psychiatry department with complaints of decreased social interaction, poor self-care, talking to self, disturbed sleep, and decreased food intake for the past 20 days. He recently joined a meditation class two months ago, and the symptoms emerged after the practice of deep meditation for long hours during the past 30 days at the meditation classes. He gradually started avoiding people at home and the workplace. He spent most of his time in meditation classes or his room. His food intake and sleep gradually decreased, and he started smiling without reason; sometimes, he even started muttering. There was no history of medical, surgical, or psychiatric illness in the past. He has an occasional history of alcohol intake, which also stopped after 30 days. Premorbidly, he was stable and well-adjusted. On detailed MSE, his psychomotor activity decreased, he had a shallow affect, he gestured to himself, and he had a second-person auditory hallucination. His physical and neurological examinations were within normal limits. His routine blood investigations, i.e., ECG, CT brain, and EEG, did not show any significant abnormalities. He was diagnosed with an acute and transient psychotic disorder and was admitted to the psychiatric wards. His symptoms decreased with oral risperidone 4 mg over 10 days, he was continuously taking this medication for six months, after which the dose was reduced to 3 mg and treatment stopped after one year.

## Results

Here, we examine the impact of meditation on mental health. We have a detailed evaluation of seven cases. After a detailed analysis, we found that our cases had psychiatric symptoms that were precipitated after meditation. Thus, meditation might be related to the etiology of psychosis in patients doing meditation, as all of our patients have a prior history of meditation just before the onset of symptoms, as shown in the table below. Moreover, as shown in Table 1, only one patient has a past history of psychiatric disorders. Thus, meditation might be one of the precipitating factors.

**Table 1:** Case presentation and impact of meditation.

Case no.	Age	Sex	History of psychiatric illness	History of substance abuse	Family history	Duration of meditation	Diagnosis
1	26 years	Female	No	No	No	1 month	Mania
2	30 years	Female	No	No	No	2 years	Schizophrenia
3	27 years	Male	No	No	No	6 months	Acute and transient psychotic disorder
4	43 years	Male	1 episode mania	No	No	10 days	BPAD ‘Mania’
5	38 years	Female	No	No	No	25 days	Acute and transient psychotic disorder
6	21 years	Male	No	No	No	1 week	Depersonalization-derealization syndrome
7	44 years	Male	No	Occasional alcohol	No	1 month	Acute and transient psychotic disorder

## Discussion

Meditation leads to complex neurochemical changes in the brain [11]. These neurochemical changes include an increase in dopamine, serotonin (5-hydroxytryptamine or 5-HT), melatonin, acetylcholine, glutamic acid, N-acetyl aspartate amino acid, glutamate, gamma-aminobutyric acid, and dimethyltryptamine (DMT), as well as a decrease in noradrenaline, cortisol, and corticotropin-releasing hormone at the central level [12]. It also increases cerebral blood flow in brain areas like the frontal lobe, pre-frontal cortex (PFC), thalamus, hippocampus, hypothalamus, and cingulate gyrus [13,14]. The altered states of consciousness seen during certain meditation practices are principally due to transient PFC deregulation. Similar findings are seen in patients with schizophrenia regarding synaptic connectivity and the neuronal thickness of the PFC [15]. There are a number of theories regarding how meditation might cause psychotic episodes. Some of the proposed mechanisms in relation to raised 5-HT2 receptor activation, elevated 5-methoxy DMT, increased NAAG, and increased dopamine. The mechanisms include the 5-HT inhibition of the lateral geniculate body, the hallucinogenic effects of DMT, the dissociative hallucinogenic effects of NAAG, and the action of increased dopamine in the temporal lobe.

Many studies show that meditation and yoga have positive effects on schizophrenia patients [16]. However, still, few authors believe that meditation should be contraindicated in those patients who are suffering from psychiatric illness. Sharma et al. stated that, as compared to mindfulness practice, transcendental meditation can be more prone to psychosis [17,18].

The psychiatric symptoms triggered by meditation closely resemble experiences by spiritually advanced personalities described in ancient texts. This prompts the inquiry of whether these experiences should be classified as psychiatric or spiritual. One possible explanation could be that both the "spiritually advanced people" and the "individuals with meditation-induced psychiatric manifestations" undergo alteration in their sense of "self," but the psychiatric manifestations result from a disruption of personality, whereas the spiritual experiences involve graded, methodological, and systematic thinning out of the selfish ego, allowing the individual consciousness to merge into universal consciousness [19].

ln the condition of dissociation, intense focus on detachment from thoughts and sensations can mimic or worsen dissociative states, particularly in vulnerable individuals. while in case of emotional dysregulation, meditation can unearth buried emotional trauma, overwhelming the individual if coping mechanisms are inadequate. For affective changes, practices encouraging high-energy states or euphoria might disrupt mood regulation, triggering manic or hypomanic episodes in predisposed individuals. In the case of depersonalization, focusing heavily on observing thoughts and feelings without attachment may deepen feelings of unreality or disconnection. Similarly, in obsessive thinking, the meditation's emphasis on awareness and repetitive focus may unintentionally amplify obsessive patterns. In the case of psychosis, overintense or prolonged meditation can blur boundaries between reality and imagination, triggering psychotic episodes in vulnerable individuals. The explanation of social isolation is overprioritizing meditation over social activities, which will reduce support systems, worsening loneliness and depression.

Abraham Maslow, who first inspired humanistic psychology, also noticed that people involved in different spiritual practices, like meditation, might be facing unexpected experiences that may manifest in varying degrees of symptoms. They called this problem, depending on its intensity, “spiritual emergence” or “spiritual emergency" [20].

Patients in our case series who experienced psychotic episodes were precipitated by meditation, and they had attended intensive meditation retreats rather than practicing brief mindfulness interventions. The causality in meditation-induced psychosis or other psychiatric issues is that, except in one case; the rest of the six cases had no history of any psychiatric illness. All of our patients were involved in intense meditation practice, and five out of seven have a history of one month to two years of deep and intense meditation. Moreover, their meditation practices increased just before the onset of psychiatric symptoms. The other two cases also started experiencing psychotic symptoms within a few days of starting meditation. Moreover, the symptoms show a response on low doses of medication once the meditation was stopped by the patient, suggesting the linkage of symptoms with meditation. Similar cases of meditation-induced psychosis were also reported in the literature in the past [8,21,22]. The literature also shows that persons practicing unsupervised deep meditation for a long period of time, coupled with sensory deprivation, are linked with the onset of psychosis [22,23].

This case series also has certain limitations. First, the temporality between meditation with psychosis cannot be proven confidently as there are multiple confounding factors like sensory deprivation, loss of sleep, and fasting, which we also have to consider precipitate psychosis [24]. Second, the corroborative history is obtained only from the patient and attender, not from their meditation trainer/expert. Hence, the information presented is primarily based on the caregivers' and patients’ versions, so their right way of practicing meditation is not clear. Moreover, only the short to medium course duration of the illness is available, so the long-term course and association with meditation are not available currently.

## Conclusions

Our case series sheds light on the fact that, beyond the often-celebrated and well-documented beneficial effects of meditation, there exist unexplored and potentially harmful manifestations. Our findings underscore the importance of approaching meditation with a nuanced perspective, recognizing its dual nature as a double-edged sword. While meditation, when practiced under the guidance of trained and qualified experts, can contribute positively to mental well-being, our observations highlight the risks associated with unsupervised and intense meditation. The development of psychiatric manifestations in such cases emphasizes the need for caution and the imperative of undertaking meditation practices under the supervision of qualified meditation experts. The idea that meditation is a formidable instrument that requires responsible and educated usage is further supported by the realization that, as we traverse the complex terrain of the mind, it is just as important to comprehend the possible risks of meditation as it is to recognize its advantages.

## Figures and Tables

**Figure 1:**
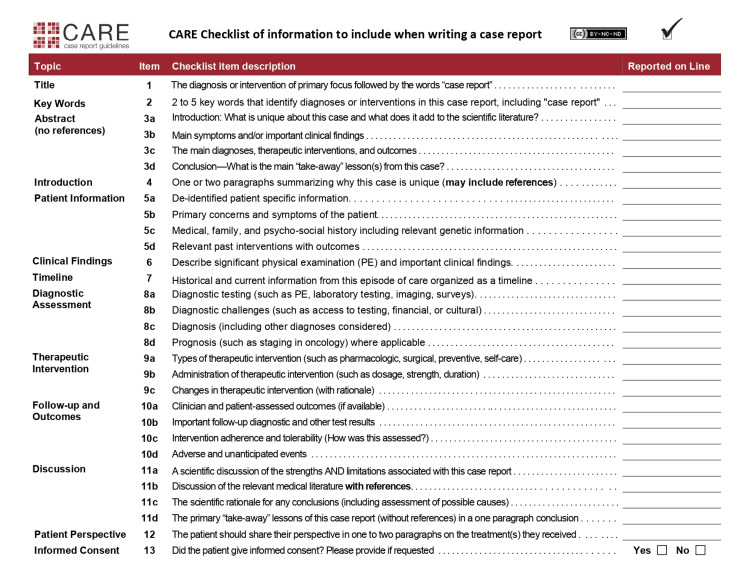
CARE (case report) checklist
